# Impacts of antenatal nursing interventions on mothers’ breastfeeding self-efficacy: an experimental study

**DOI:** 10.1186/s12884-019-2701-0

**Published:** 2020-01-06

**Authors:** Safiya Sabri Piro, Hamdia Mirkhan Ahmed

**Affiliations:** 1 College of Nursing, University of Duhok, Duhok, Kurdistan Region Iraq; 2 College of Nursing, Hawler Medical University, Erbil, Kurdistan Region Iraq; 3 College of Health Sciences, Hawler Medical University, Erbil, Kurdistan Region Iraq

**Keywords:** Breastfeeding self-efficacy, Knowledge, Attitude, Exclusive breastfeeding

## Abstract

**Background:**

A considerable amount of research demonstrates how breastfeeding self-efficacy significantly influences breastfeeding outcomes. The aim of this study was to evaluate the role of nursing intervention on mother’s breastfeeding self-efficacy.

**Methods:**

In this experimental investigation, 130 pregnant women who attended a primary health care centre were randomly assigned to the experimental (*n* = 65) or control (*n* = 65) groups. The experimental group received two 60–90 min group breastfeeding educational sessions based on the breastfeeding self-efficacy theory along with routine care. Mothers’ knowledge, attitudes, prenatal and postnatal self-efficacy towards the breastfeeding were compared between both groups. The Iowa Infant Feeding Attitude Scale measured the attitudes. Prenatal Breastfeeding Self-Efficacy Scale measured the self-efficacy during pregnancy and Breastfeeding Self-Efficacy-Short Form measured the self-efficacy in postnatal period.

**Results:**

Breastfeeding self-efficacy during pregnancy and following two months of delivery in the experimental group was significantly higher. The experimental group had a higher level of knowledge and attitude in comparison with subjects in the control group. In addition, the mothers who breastfed exclusively had higher levels of postnatal self-efficacy in both experimental and control groups compared to formula feeding women (52.00 vs. 39.45 in the control and 57.69 vs. 36.00 in the experimental subjects; *P* < 0.001).

**Conclusion:**

The present investigation suggests that antenatal breastfeeding education is an effective way to increase the level of breastfeeding self-efficacy, which increases exclusive breastfeeding practice.

## Background

Breastfeeding (BF) is an art, and human milk has no another exact alternative for feeding babies. Breastfeeding assists in developing an indelible connection between the mother and baby [1]. Recently, the promotion of BF has increased by health systems in line with World Health Organization (WHO) and United Nations International Children’s Emergency Fund (UNICEF) policies, and there have been numerous efforts to support, promote and retain BF [2]. Despite these efforts on BF, globally only 44% of infants initiate BF within the first hour after birth and only 40% of all infants under six months of age are exclusively breastfed. And only 45% of children are still BF at two years of age [3].

Although, there is a lack of recent data on infant and young child feeding practices in Iraq, a presented report by International Baby Food Action Network (IBFAN) claimed low rates of early initiation of BF (43%), exclusive BF under 6 months (20%), BF at 2 years (23%) and high rates of bottle feeding (64%) in Iraq [4].

Maternal self-efficacy in BF is one of the potentially modifiable factors which is consistently linked with positive BF outcomes [5]. The literature declares that “mother’s inadequate breastfeeding self-efficacy (BSE),” “incompetency of BF services”, and “family’s neglect to breast milk” are challenges and barriers to BF promotion [6]. Breastfeeding self-efficacy refers to a mother’s confidence in her capability to breastfeed her infant [7]. Mother’s self-efficacy is an essential variable in BF outcomes as it predicts: (1) whether a mother chooses to breastfeed as the desired infant feeding method. (2) how much effort she will expend during BF. (3) whether mother will persevere in her attempts until mastery is achieved. (4) whether she has self-enhancing or self-defeating thought patterns. (5) how she emotionally responds to breastfeeding difficulties [7].

Educational interventions have an impact on the health of the pregnant woman as well as, on the health and wellbeing of the next human generations. Antenatal BF education is beneficial in preparing women for effective BF by promoting their confidence level, knowledge, and skills [8].

Invariably, nurses guide and assist women throughout their pregnancy and puerperal period. She also plays a vital role in health education programs during perinatal care [9]. A nurse can encourage the advancement of BF by providing BF teaching and positive support before birth and after hospital discharge [10].

Few studies concentrated on the effect of antenatal education on BSE of Kurdish women in the Kurdistan region of Iraq. Therefore, the researchers attempted to evaluate the effectiveness of the nursing intervention on BSE, knowledge and attitude of a sample of women in Iraqi Kurdistan. The authors hypothesized that the BSE of the women who receive a nursing intervention program would be higher compared to those women who do not receive the nursing intervention program.

## Methods

### Study design and subjects’ recruitment

**Fig. 1:**
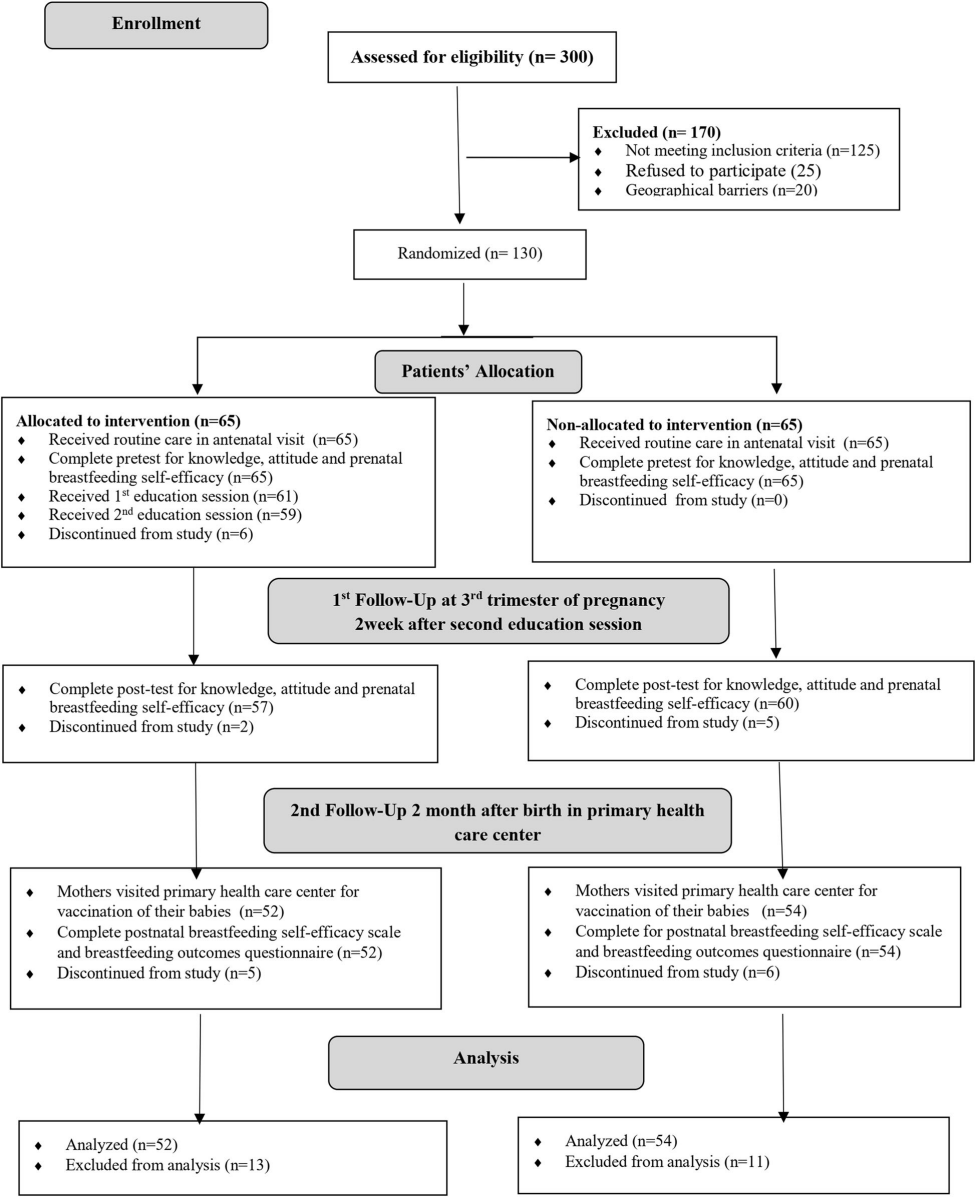
Flow diagram of participants’ recruitment

An experimental study was conducted on 130 pregnant women who attended a primary health care centre (PHCC) for antenatal care, medical check-up, and vaccination. The PHCC is one of the biggest health setting in Erbil city and provides primary health care for a significant target population. The participants were selected from a total of 300 pregnant women who met all eligibility criteria of the study. The study was conducted from October 2017 until July 2018. The subjects were assigned either into intervention (*n* = 65) or control (n = 65) group in a random way through generating a random digit number by Microsoft Excel 2013. The subjects were Kurdish speakers and pregnant women who enrolled in the maternal care unit of PHCC, from 30th to 38th weeks of their gestational age, had a normal pregnancy without complications, expected to have a singleton, were full term and normal new-born, either by vaginally or caesarean section. The included subjects agreed to participate in the study and were ready to be followed-up by the researcher during the study period. Those pregnant subjects with the following conditions were excluded from the study:

1. Medical issue could significantly impact on BF
2. Had inverted nipple
3. Expected to deliver a preterm new-born or a new-born with complications and/or congenital abnormality (diagnosed based on the medical and clinical examinations)
4. Intended to exclusive formula feed
5. Dropped during the study time (Fig. 1).

### Study tools for data collection

The data set of the study was collected, measured, and recorded in a pre-designed investigator-administered questionnaire. The questionnaire had five parts as following:

- **Part A:** The demographic characteristics of the subjects were collected prior to implementation of the BF education. The general information was based on: age (years), age at marriage (years), education (years), gestational age (weeks), occupation (categorized as employed/housewife), family type (categorized as nuclear/ extended), gravida (primigravida /multigravida), para (primiparous/multiparous), and abortion (non-aborted/aborted).
- **Part B:** A questionnaire format was developed by the researcher after an extensive literature review regarding BF and according to the recommendations of WHO and UNICEF. The questionnaire of mothers’ knowledge toward BF consisted of 21 items that were divided into three sections. Items were scored as one when answered correctly or zero when answered incorrectly. The total score of the scale ranged from 0 to 21 points, with a higher score indicating a higher degree of maternal BF knowledge. The **first section** had 8 question items relating to the benefits of BF for the infant. (1) Provides perfect and healthy nutrition. (2) Protects against infection and illnesses. (3) Promotes bonding between mother and child. (4) Breastfeed baby is rarely constipated. (5) Protect baby from obesity. (6) Strengthen child’s bones. (7) Enhances development. (8) Enhances intelligence. The **second section** consisted of 6 question items about the benefits of BF for the mother (1) Leads to uterine involution (2) Decreases incidence of breast cancer (3) Decreases incidence of ovarian cancer (4) Provides emotional satisfaction to the mother (5) Loses pre-pregnancy weight faster (6) Spaces birth. The **third section** included 7 question items recommended by WHO and UNICEF. The question items were; (1) Do you know about early skin-to-skin contact? (2) When the mothers have to initiate the BF after childbirth? (3) Should colostrum be given to the baby? (4) Is it necessary to give prelacteal feeds to baby? (5) Up to which age child should receive exclusive breast milk? (6) At which age the complementary food should be introduced? (7) What is the optimal time for weaning the infant?
- **Part C:** In this part of the questionnaire, the mothers’ attitudes were measured using the Iowa Infant Feeding Attitude Scale (IIFAS). IIFAS was developed by Mora and Russell (1999) for assessment of mothers’ attitude toward breastfeeding. IIFAS is a valid and reliable analytical tool with Cronbach’s alpha ranging from 0.85 to 0.86. IIFAS consists of 17 attitude questions rated by a 5-point Likert ranging from 5 (strongly agree) to 1 (strongly disagree). According to this scale, approximately one-half of the items are favourable to BF, and the remaining favourable to formula feeding. Items favouring formula feeding are reverse scored and a total attitude score computed via an equally weighted sum of responses. Total attitude scores range from 16 (indicating positive attitudes towards artificial feeding) to 80 (reflecting positive attitudes towards BF). The last item of the scale was “A mother who occasionally drinks alcohol should not breastfeed her baby” did not receive any score in this study, as all subjects of the study were non-alcoholics [11].
- **Part D:** The prenatal BSE of study participants were measured through Prenatal Breastfeeding Self-Efficacy Scale (PBSES). The scale was developed and provided evidence for internal consistency (Cronbach’s alpha = 0.89), as well as validity by Wells et al. (2006). This instrument was designed to a 20-item Likert scale method with responses ranging from 1 (not sure) to 5 (completely sure). Possible overall scores range from 20 to 100, with higher scores indicative of greater BSE of mothers during pregnancy [12]. Items 11 and 20 were slightly modified to be compatible with Kurdish culture.
- **Part E:** Postnatal BSE of the subjects two months after birth was measured by Breastfeeding Self-Efficacy Scale-Short Form (BSES-SF). The Cronbach’s alpha coefficient for the BSES-SF was 0.97. It consists of 14 positive statement inquiries as developed by Dennis in 2003 to measure BSE. Participants were asked to rate their agreement with the statements based on a 5-point Likert-type scale. A response of ‘1’ indicates that the participant strongly disagrees or not at all confident and response of ‘5’ indicates that the participant strongly agrees or very confident with the statement. The scores are then summed to produce a possible range from 14 to 70, with higher scores indicating higher levels of BSE [13].

The questionnaire of mothers’ BF knowledge, IIFAS, PBSES and BSES-SF were translated into Kurdish language by a translation office that is authorized by Ministry of Justice of Kurdistan/Iraq. Then the Kurdish version was reviewed by four academic experts (two in maternity nursing and two in paediatric nursing) whose mother tongue is Kurdish language, but they are fluent in English language. The one by one translation items of these scales were discussed by the experts. They were reconciled to a Kurdish version.

### Nursing intervention

A direct interview technique was used by the researcher to gather the required information before the intervention. The investigators reviewed the daily appointment schedule of pregnant women in antennal care unit in Mala Afendy PHCC in Erbil city. Determination of eligible participants was based on the selection criteria. Verbal consent was obtained after explaining the purpose of the study and the process of intervention. The subjects in the intervention group were masked to the fact that they would be compared to a control group in the health centre. They were allocated either into the intervention or the control group randomly upon agreement. After taking the demographic data, a baseline of mother’s knowledge, attitude and prenatal BSE scale was completed for both groups as a pre-test by the second researcher. Researchers made a schedule to demonstrate date and time of meetings for two educational sessions just for the participants in the intervention group. Post-test of knowledge, attitude and prenatal BSE scale was taken for both intervention and control group two weeks after the pre-test. All participants in the intervention group were called and educated in small groups. If the participants could not attend according to the schedule in the educational sessions, another appointment was arranged by the investigator with another group.

The intervention was an antenatal BF educational program which was developed after reviewing an extensive literature and standards of BF education that was based on the BSE framework of Dennis (1999). Dennis theorized the role of self-efficacy in breastfeeding in order to explain and predict behaviour in her framework [7]. The theory of self-efficacy suggests that four factors can influence the level of self-efficacy of a person. These are personal achievements, vicarious experiences, verbal persuasion, and psychological and affective states [7, 14].

Also, for the educational session, the required information on BF aspects with related images were collected in a forty page booklet in local language by researchers. The information were simple anatomy and physiology of BF, the benefits of BF for both infant and mother, initiation of BF, benefits of skin-to-skin contact, common position of BF. Moreover, other information was the baby attachment to the breast, signs of effective sucking, methods of milk expression, successful BF tips and common problems that lactating mothers encountered during the initial stages of BF, and how these challenges were overcome.

The PHCC did not present the official antenatal educational services on BF to the participants. Both groups received routine antenatal care which included checking weight, blood pressure, urine for protein and sugar, and foetal heart rate. These tests were delivered by the medical health care provider in the PHCC. In addition to the routine care, participants in the intervention group received a BF booklet and two BF education sessions in small groups of four to six participants with two days interval, each session lasting for 60–90 min. During these sessions, the researcher explained all contents of the booklet to the participants. In addition, some related videos were downloaded on a laptop and were displayed for approximately 15 min to participants to facilitate the educational process. The educational sessions were open to the discussion of the participants’ issues on BF. We gave an opportunity to the subjects who did not understand the information given in the face to face counselling, booklet, or videos to make a contact with the researcher for further clarification through a phone counselling. Two months after childbirth at the time of vaccination of new-born baby, all participants in both groups were again interviewed by filling out the postnatal BSES-SF and enquired on infant feeding status.

### Statistical analysis

The collected data were coded, entered, verified, and analyzed using the Statistical Package for Social Sciences version 25:00 (SPSS 25:00; IBM Corp; USA).

The categorical and numerical characteristics of mothers were present in frequency (percentage) and mean (Sta. Deviation), respectively. The homogeneity of baseline information between the experimental and control groups was examined in the Pearson Chi-Square test or independent t-test. The difference of knowledge and attitude items between both groups were determined in Pearson Chi-square tests and independent t-test, respectively. The difference of prenatal and postnatal BSE items between the study and experimental groups were determined in independent t-test, respectively. The differences between postnatal BSE and exclusive BF were examined in independent t-test. Binary logistic regression was performed for predictors of exclusive BF in the experimental group. The null hypothesis was rejected in a *P*-value of less than 0.05.

### Ethical considerations

The protocol of the study was approved by the Scientific and Ethical committee of College of Nursing/ Hawler Medical University (registration number: 9 in 16/3/2016). Formal permission was given by the health care centre. The level of intervention that was applied to the participants in the experimental group was considered to be safe. Data were collected from mothers who agreed to participate in the study. The required information was explained to all participants. Participation was voluntary, anonymous and their right to withdraw from the study was protected. Verbal consent was obtained from all study participants. Also, the confidentiality of all personal information of the study sample was protected throughout the study duration. Participants’ consent was taken to publish the information reported in the present trial.

## Results

Baseline information of the participants in the experimental and control groups was checked before the analysis of the impact of antenatal education. The analysis identified that both control and experimental groups were similar in age (26.80 vs 26.38 years, *P* = 0.724); age at marriage (20.26 vs. 20.94 years, *P* = 0.360); education (7.58 vs. 8.25 years, *P* = 0.472); gestational age (33.94 vs. 33.97 weeks, *P* = 0.948); occupation (housewife: 92.3% vs. 86.2%, *P* = 0.258); Gravida, Para and Abortion (*P* > 0.05) and lactation history (45.6% vs. 42.6%, *P* = 0.749). The only difference was the type of family (nuclear family 73.8% vs 56.9%, *P* = 0.043) (Table 1).

**Table 1:** Comparaison of mothers’baseline information

Subjects’ characteristics	Control (*n* = 65)	Experimental (*n* = 65)	*P*-Value (Two-Sided)
Age (Years)	26.80 (6.60)	26.38 (6.80)	0.724*
Age at marriage (Years)	20.26 (4.07)	20.94 (4.32)	0.360**
Education (Years)	7.58 (5.38)	8.25 (5.08)	0.472*
Gestational Age (weeks)	33.94 (2.62)	33.97 (2.78)	0.948 Independent t-test
Occupation			
Employed	5 (7.7)	9 (13.8)	0.258*
Housewife	60 (92.3)	56 (86.2)	
Family Type			
Nuclear	48 (73.8)	37 (56.9)	0.043*
Extended	17 (26.2)	28 (43.1)	
GPA			
Gravida			
Primigravida	31 (47.7)	35 (53.8)	0.483*
Multigravida	34 (52.3)	30 (46.2)	
Para			
Primiparous	36 (55.4)	40 (61.5)	0.477*
Multiparous	29 (44.6)	25 (35.8)	
Abortion			
Non-Aborted	50 (76.9)	54 (83.1)	0.380*
Aborted	15 (23.1)	11 (16.9)	
Lactation history	26 (45.6)	23 (42.6)	0.749*

*Chi-Squared and ** Fishers’ Exact tests were performed for statistical analyses

The homogeneity of knowledge, attitudes, and prenatal BSE of the participants were assessed after randomization process (pre-test) by the same measurement tools for both study groups. The assessment of the knowledge showed a non-significant difference (*p* > 0.05) between two groups. In addition, the total degree of prenatal BSE between the experimental and control groups; 53.57 (SD: 9.53) vs. 53.88 (SD: 9.63); *P* = 0.855 and their contents were not different significantly (*P* > 0.05). The total level of attitudes between the two study groups were not different substantially; 58.02 (SD: 4.06) vs. 58.35 (SD: 4.20); *P* = 0.641 and their contents (*P* > 0.05).

Two week after completing the educational intervention a post-test of knowledge, attitudes, and prenatal BSE was taken for both groups of the present study, the results indicated that the total number of correct knowledge answers (21 knowledge items) after intervention were significantly higher (780, 65.15%) in the experimental group compared to those items answered correctly by control subjects (421, 33.41%). As well, the total attitude score of the subjects in the experimental group was significantly higher (Mean [M]: 62.88, SD: 3.35) compared to the control group (M: 58.57, SD: 4.25), *P* < 0.0001 (Table 2).

**Table 2:** Comparison of mothers’ overall knowledge and attitude towards BF after intervention (post-test)

Subjects’ Knowledge and attitude	Control (*n* = 60)	Experimental (*n* = 57)	*P*-Value (Two-Sided)
Total correct answers	421 (33.41)	780 (65.15)	< 0.0001*
Total attitude	58.57 (4.2)	62.88 (3.4)	< 0.0001**

*Pearson Chi-Square and **Independent t-test were performed for statistical analyses

After intervention the total prenatal BSE level was substantially higher in the experimental group (M: 70.84, SD: 8.68) compared to the control group (M: 55.02, SD: 9.49), *P* < 0.0001. The significant higher scores were found in all items of prenatal BSE in the experimental group compared to those in the control group (*P* < 0.001), (Table 3).

**Table 3:** Comparison of mother’s prenatal BSE after intervention (post-test)

Prenatal BSE items	Control (*n* = 60)	Experimental (*n* = 57)	*P*-Value (Two-sided)
I can make time to breastfeed my baby even when I feel busy.	2.82 (1.03)	3.42 (1.00)	0.002
I can breastfeed my baby even when I am tired.	2.65 (1.15)	3.47 (1.02)	< 0.0001
I can schedule my day around the breastfeeding of my baby.	2.52 (0.93)	3.16 (1.07)	0.001
I can breastfeed my baby when I am upset.	2.53 (1.07)	3.42 (1.07)	< 0.0001
I can breastfeed my baby even if it causes mild discomfort.	2.65 (1.12)	3.19 (1.09)	0.009
I can use a breast pump to obtain milk.	2.22 (1.08)	2.96 (1.13)	< 0.0001
I can prepare breast milk so others can breastfeed my baby.	2.37 (1.25)	3.05 (1.09)	0.002
I can find out what I need to know about breastfeeding my baby.	2.45 (0.91)	3.53 (.80)	< 0.0001
I can find the information I need about problems I have breastfeeding my baby.	2.38 (0.85)	3.42 (.87)	< 0.0001
I know who to ask if I have any questions about breastfeeding my baby.	2.43 (0.98)	3.65 (.77)	< 0.0001
I can call a physician if I have problems breastfeeding.	1.93 (0.97)	2.81 (1.01)	< 0.0001
I can talk to my healthcare provider about breastfeeding my baby.	2.78 (0.98)	3.82 (.71)	< 0.0001
I can breastfeed my baby when my family or friends are with me.	2.70 (1.18)	3.58 (1.21)	< 0.0001
I can breastfeed my baby around people I do not know.	2.12 (1.14)	3.28 (1.26)	< 0.0001
I can breastfeed my baby when my husband is with me.	3.37 (1.03)	4.19 (0.79)	< 0.0001
I can breastfeed my baby without feeling embarrassed.	2.47 (1.02)	3.12 (0.91)	< 0.0001
I can choose to breastfeed my baby even if my husband does not want me to.	3.38 (0.92)	4.25 (0.76)	< 0.0001
I can choose to breastfeed my baby even if my family does not want me to.	4.03 (0.82)	4.40 (0.56)	0.005
I can talk to my husband about the importance of breastfeeding my baby.	3.92 (0.85)	4.35 (0.64)	0.002
I can breastfeed my baby for two years.	3.30 (1.03)	3.75 (0.87)	0.001
Total prenatal BSE	55.02 (9.49)	70.84 (8.68)	< 0.0001

Independent t-test was performed for all statistical analyses. The numbers are in mean (standard deviation).

The postnatal BSE was measured after two months of delivery in both groups. The findings showed that the total postnatal BSE mean score was substantially higher in the experimental groups (M: 53.98, SD: 8.50) compared to the control group (M: 43.41, SD: 8.12), *P* < 0.0001. Generally all postnatal BSE items score were significantly higher in the experimental group compared to the control group. The subjects in the experimental group agreed with having the ability of achieving the following statement: (1) Determine their baby was getting enough milk (M: 3.87, *P* < 0.0001). (2) Cope successfully with breastfeeding as with other challenging tasks (M: 3.79, *P* < 0.0001). (3) Breastfeed their baby without using formula as a supplement (M: 3.96, *P* < 0.0001). (4) Ensure their baby was properly latched on for the whole feeding (M: 3.77, *P* < 0.0001). (5) Manage the BF situation to their satisfaction (M: 3.83, *P* < 0.0001). (6) Manage to breastfeed even if their baby was crying (M: 3.79, *P* < 0.0001). (7) Kept wanting to breastfeed (M: 4.04, *P* = 0.039). (8) Breastfeed comfortably with presence of their family members (M: 3.92, *P* < 0.0001). (9) Were satisfied with their BF experience (M: 4.08, *P* < 0.0001). (10) Deal with the fact that BF can be time-consuming (M: 3.87, *P* = 0.013). (11) Finish feeding their baby on one breast before switching to the other breast (3.69, *P* = 0.001). (12) Continue to breastfeed their baby for every feeding (M: 3.71, *P* < 0.0001). (13) Manage to keep up with their baby’s BF demands (M: 3.85, *P* < 0.0001). (14) Tell when their baby was finished BF (M: 3.83, *P* = 0.001), (Table 4).

**Table 4:** Comparison of mothers’ postnatal BSE two months after birth

Postnatal BSE items	Control (*n* = 54)	Experimental (*n* = 52)	*P*-Value (Two-Sided)
1. Determine that my baby is getting enough milk	3.13 (0.97)	3.87 (0.79)	< 0.0001
2. Successfully cope with breastfeeding as I have with other challenging tasks	2.76 (1.23)	3.79 (0.83)	< 0.0001
3. Breastfeed my baby without using formula as a supplement.	2.39 (1.24)	3.96 (1.03)	< 0.0001
4. Ensure that my baby is properly latched on for the whole feeding.	3.04 (0.85)	3.77 (0.92)	< 0.0001
5. Manage the breastfeeding situation to my satisfaction.	2.91 (1.05)	3.83 (0.83)	< 0.0001
6. Manage to breastfeed even if my baby is crying	3.07 (0.95)	3.79 (0.94)	< 0.0001
7. Keep wanting to breastfeed.	3.74 (0.81)	4.04 (0.66)	0.039
8. Comfortably breastfeed with my family members present.	3.19 (0.80)	3.92 (0.68)	< 0.0001
9. Be satisfied with my breastfeeding experience.	3.07 (1.06)	4.08 (0.65)	< 0.0001
10. Deal with the fact that breastfeeding can be time-consuming.	3.52 (0.75)	3.87 (0.66)	0.013
11. Finish feeding my baby on one breast before switching to the other breast.	3.06 (0.88)	3.69 (1.04)	0.001
12. Continue to breastfeed my baby for every feeding.	3.11 (0.97)	3.71 (0.89)	< 0.0001
13. Manage to keep up with my baby’s breastfeeding demands.	3.13 (0.78)	3.85 (0.87)	< 0.0001
14. Tell when my baby is finished breastfeeding	3.30 (0.84)	3.83 (0.76)	0.001
Total postnatal BSE	43.41 (8.12)	53.98 (8.50)	< 0.0001

Independent t-test was performed for all statistical analyses. The numbers are in mean (standard deviation)

The comparison of total postnatal BSE two months following of delivery showed that its level was higher in those mothers who exclusively breastfed compared to those subjects who had partially breastfeed and formula fed in both intervention and control groups (*P* < 0.001), (Table 5).

**Table 5:** Comparison of mothers’ total postnatal BSE with infants feeding status (two months after delivery)

Postnatal BSE	Control		*P*-Value (Two -Sided)	Experimental		*P*-Value (Two-Sided)
Infants feeding status	n	Mean (SD)		n	Mean (SD)	
Exclusively BF	13	52.00 (10.5)	< 0.001	29	57.69 (4.86)	< 0.001
Partially BF	30	41.46 (6.17)		19	52.11 (5.16)	
Formula feeding	11	39.45 (4.55)		4	36.00 (16.15)	

Pearson Chi-squared test was performed for statistical analysis

The Logistic Regression Analysis of the study showed that higher postnatal BSE predicted a higher level of exclusive BF in the experimental study groups; OR: 0.661 (95% CI: 0.50–0.87); *P* = 0.004 (Table 6).

**Table 6:** Predictors of exclusively breastfeeding in the experimental group

Predictors	Dependent variable: Exclusively and noon-exclusively breastfeeding in the experimental group						
	B	S.E.	Wald	*P*-Value	OR	95% C.I. OR	
						Lower	Upper
Age (Years)	−0.343	0.190	3.255	0.071	0.710	0.49	1.03
Age at marriage (Years)	0.269	0.197	1.867	0.172	1.308	0.89	1.92
Education level	2.122	1.449	2.145	0.143	8.346	0.49	142.78
Occupation	−1.524	1.586	0.923	0.337	0.218	0.01	4.88
Type of family	0.710	1.136	0.390	0.532	2.034	0.22	18.85
Gravida	−3.462	1.930	3.217	0.073	0.031	0.001	1.38
Para	3.594	2.161	2.765	0.096	36.367	0.53	2513.92
Lactation history	1.292	1.647	0.616	0.433	3.641	0.14	91.80
Total attitude	0.281	0.207	1.831	0.176	1.324	0.88	1.99
Total postnatal BSE	−0.413	0.142	8.441	0.004	0.661	0.50	0.87

Binary logistic regression was performed for statistical analysis

## Discussion

### Breastfeeding self-efficacy

Self-efficacy is crucial in BF and it is regarded to be a determining parameter in relation to choosing BF method and level of compliance in solving BF problems [15, 16].

The results of the present study were compatible with the hypothesis that BSE can be increased through prenatal nursing intervention.

Many researches have supported prenatal BF interventions as being effective in increasing BSE regardless of the types of educational intervention [16–18]. A recent meta-analysis from Canada investigated the effect of education or support based interventions on improvement of BSE. The interventions were implemented in the postpartum, prenatal or perinatal period. The results indicated that the mothers in the intervention groups had significantly higher BSE score compared to the mothers in the control groups [15].

In contrast with our results, in an evidence-based practice project, eight nulliparous pregnant women who were 14 to 18 years of age and were in high school, participated in a pre and post-intervention study. The results reported no significant differences in prenatal BSE scores in pre and post-intervention (an antenatal educational intervention) [19].

Probably this difference between the results of our study and that project could be interpreted as following: Important reason is low sample size (eight participants compared to 130 in our study is very low), which did not support the achievement of statistical significance. In addition, design of study has a great importance, as our study design is a case - control study while, the mentioned study was a pre and post-intervention design. Therefore, there may be a confounding factor affected the BSE at pre-test, whereas control group could have solved this problem. The other reason for the difference reflected in the characteristics variation of participants in both study. For instance, all participants in that project were nulliparous, so they did not experience breastfeeding. While approximately half of the subjects in our research had at least one child and more than 40% of them had lactation history. In the other hand, all mothers in the mentioned project were adolescent students, while the majority of participants in our study were housewives and nearly at mean age of 26 years. Furthermore a study from Portugal found an association between BSE and women’s parity, educational level, occupational status, and previous breastfeeding experience [15, 20].

Aguirre, et al. (2018), noted that the effect of the prenatal intervention on BSE could be changed over time point. In this regard, they did not find a significant difference between intervention and control group at baseline or during the early days postpartum. However, differences in self-efficacy scores were considerable at week 6 and months 3 and 6 [21].

Literature supports positive effectiveness of antenatal education on increasing BSE but, sometimes the context and circumstances may impact on the effectiveness of the interventions. In this regards, a Japanese study explored impact of a self-efficacy intervention on BSE and exclusive BF and assessed the difference in effect by hospital-routine type. The eligible pregnant women at third trimester were enrolled from non-Baby-Friendly Hospitals (nBFH) and “Baby-Friendly”-certified hospitals (BFH). They were assigned to either the intervention group or the control group. A breastfeeding self-efficacy workbook was provided only for the participants in the intervention group from both types of hospitals. In BFHs, the intervention improved both BSE and exclusive BF at four weeks postpartum. But, in nBFHs, no positive result was observed on BSE or on the exclusive BF rate through four weeks postpartum [22]. Therefore, more attention should be paid to mothers’ conditions and their limitations concerning time and place of their education [23].

In the present study postnatal BSE was found to be a predictor of exclusive BF in experimental group but not regarding other variables such as age, age at marriage, educational level, occupation, type of family, gravidity, parity and lactation history. Although many studies in the Kurdistan region were done on knowledge and attitude of breastfeeding, but the present study is the first one which examined the BSE of Kurdish mothers through nursing intervention.

### Exclusively breastfeeding

The findings of the present study suggest that the prenatal nursing intervention was effective in increasing postnatal BSE which led to enhancing the exclusive BF practice after two months of birth. In consequence, a higher postnatal BSE score associated with a higher level of exclusive BF practice in both groups of study.

Brockway et al. (2018), claimed that self-efficacy can predict BF outcomes in first and second months following birth in mothers of full-term infants, and it is a modifiable factor that can affect BF success [15, 24].

Literature proved that mothers’ breastfeeding self-efficacy and intention to BF were the most important predictors of initiation, continuing exclusive BF and length of BF during six months after birth [15, 25, 26].

Brandão et al. (2018), found that the women who had a higher BSE scores during pregnancy were BF exclusively at the first month postpartum, while women who were mixed feeding or who had stopped BF at the first month postpartum had a lower antenatal BSE scores [15, 20].

A study from Brazil, reported that the high score of BSE and the age of mothers were the protection factors to the exclusive BF [17]. Results of this study corresponds with our findings regarding the BSE as an associated factor with exclusive BF, but in our study we did not find any association between mothers’ age and exclusive BF.

A USA-based study could not find the antenatal education, postnatal BSE and BFexperience, as contributing factors of exclusive breastfeeding [18]. While our results indicated that there was a link between antenatal education and postnatal BSE with exclusive breastfeeding, but both studies agreed that the BFexperience was not a contributing factor to exclusive breastfeeding.

Globally, it is recognized that a range of cultural variables affect infant feeding practices including cultural taboos [15, 27]. But, there are few studies on cultural variables influencing BF in a developing country context [15, 28]. We do not have official data about the effect of Kurdish culture and norms on BF among Kurdish women. But according to our experiences of living in this context, generally Kurdish people encourage the women to breastfeed their infants.

### Breastfeeding knowledge and attitudes

The effect of nursing education on increasing knowledge and attitude is obvious; as the results of the present study also proves the improvement in the breastfeeding knowledge and attitude of mothers after BF education in experimental group compared to control group. Concerning the effect of knowledge on BF, several studies reported that poor maternal knowledge was a relevant risk factor for BF abandonment [29–31]. Similar with our findings, a study from Saudi Arabia reported significant differences within the intervention group in knowledge and attitude [32]. A quasi-experimental study from Iran revealed that the women who receive antenatal education have significantly better scores in terms of self-efficacy, knowledge, and attitude scores [33]. As knowing that Saudi Arabia and Iran are neighbour countries of Iraq and there is culture similarity between the countries with Islamic religious background that have favourable attitude toward breastfeeding. Another study found that introducing the prenatal education resources in the obstetrician’s waiting room significantly increased breastfeeding attitudes and knowledge among the education group [34]. It is worth mentioning that, one of the leading causes of neonatal mortality and morbidity is inadequate BF due to a lack of understanding of a mother of its importance and benefits [35]. Insufficient knowledge, poor attitude or inappropriate practice, of BF may lead to undesirable consequences for both mother and child [36]. Antenatal education is an important factor in developing BF knowledge and fostering BF skills and confidence for initiating and maintaining BF until the sixth month [29] which effects the health of the baby. Mother’s knowledge and skills can increase the rate and duration of breastfeeding and are a relevant component of effective decisions and actions related to BF [29, 37].

### Considerations on breastfeeding self-efficacy scales

There are several measurements of BSE which have been used cross-culturally with good reliability [38]. Six scales for measuring BSE were reported based on a critical review of available BSE instruments in 2015 [39]. Each of these instruments were intended to measure the BSE. The majority of the instruments were based on Bandura’s social cognitive theory directly or indirectly [39]. Three of these scales were developed to measure prenatal BSE, while others were developed to measure postnatal BSE. Breastfeeding Self-Efficacy Scale – Short Form (BSES - SF) was used more frequently than any other BSE instrument. More than forty articles have been published using this instrument [39]. Since none of the instruments can be used to measure prenatal and postnatal BSE over time, especially between anticipated self-efficacy and experience self-efficacy, the effectiveness and improvement were observed at both prenatal and postnatal period. Whereas, most available studies that measured the prenatal and postnatal BSE, used BSES -Short Form for measuring both prenatal and postnatal BSE [16, 40]. This particular instrument was not appropriate for administering to women in the prenatal period since many of the questions which assess BF confidence require a woman to have actual interaction with her baby [12, 41].

Therefore, in this study the authors decided to measure maternal BSE in the prenatal and postnatal period by two distinct instruments, that the wording of the each scales was compatible with a specific time period. The BSES-Short Form of Dennis is more suitable for postpartum which was used for measuring postnatal BSE in the present study. The scale was developed by Wells et al. was used for measuring of prenatal BSE.

### Limitations of this study

Experimental design, random group assignment and using validated instruments for measuring BF attitude and self-efficacy were strong points of the present study. However, as study sample had derived from one PHCC, this precluded us from generalizing the findings to other settings across the country. In addition, data collection were based on the mothers’ subjective views. Therefore, response bias might have occurred since participants may have tended to provide favourable responses so that they would be perceived as successful BF mothers. Consequently, the results may have been influenced by the personality and environment of the mother. Alternately, preconceived expectations of the researcher may have also affected interaction with participants.

## Recommendations

Since antenatal BF education program is a valid intervention for modifying some variables (i.e. knowledge, attitude, and BSE), results of this research could be used by nurses and health educators to guide the women in their daily practice. Consequently, there is a need to produce a standard breastfeeding educational package for pregnant mothers by health professionals. Although the present study supports the effect of intervention during pregnancy on BSE, maybe this effect could be increased by extension of interventions to the intrapartal and postpartal period. Furthermore, this type of intervention could be repeated in larger samples and with diverse populations. The study also recommends the further use of qualitative studies to gain a deeper understanding of those mothers who do not successfully breastfeed. Using BSE scale as a screening tool in the prenatal period by health care providers could identify women who need interventions during the prenatal period.

## Conclusions

Exclusive BF practise is affected by increasing BSE of mothers through antenatal nursing interventions, which also enhance the mother’s BF knowledge, and attitudes. The level of BSE is a predictor of exclusive BF practice during the first two month after birth. Since has been evidenced that there is a association between increasing exclusive BF (as an optimal infants’ nutrition) and reducing infants’ morbidity/mortality, as well provide better health status for mothers, thus a comprehensive antenatal educational programs on BF can play important role in the achievement of better health outcome for both infants and mothers.

## Data Availability

The datasets analyzed and the materials used during the current report are available from the corresponding author on reasonable request.

## Abbreviations

BF: Breastfeeding
BSE: Breastfeeding Self-Efficacy
BSES-SF: Breastfeeding Self-Efficacy Scale-Short Form
IIFAS: Iowa Infant Feeding Attitude Scale
PBSES: Prenatal Breastfeeding Self-Efficacy Scale
PHCC: Primary Health Care Centre
SPSS: Statistical Package for Social Sciences
UNICEF: United Nations International Children’s Emergency Fund
WHO: World Health Organization
